# Switching from FOLFIRI plus cetuximab to FOLFIRI plus bevacizumab based on early tumor shrinkage in *RAS* wild‐type metastatic colorectal cancer: A phase II trial (HYBRID)

**DOI:** 10.1002/cam4.7107

**Published:** 2024-04-09

**Authors:** Hiroyuki Arai, Takashi Tsuda, Yu Sunakawa, Mototsugu Shimokawa, Kohei Akiyoshi, Shinya Tokunaga, Hirokazu Shoji, Kenji Kunieda, Masahito Kotaka, Toshihiko Matsumoto, Yusuke Nagata, Takuro Mizukami, Fumitaka Mizuki, Kathleen D. Danenberg, Narikazu Boku, Takako Eguchi Nakajima

**Affiliations:** ^1^ Department of Clinical Oncology St. Marianna University School of Medicine Kawasaki Japan; ^2^ Center for Hepato‐Biliary‐Pancreatic and Digestive Disease Shonan Fujisawa Tokushukai Hospital Fujisawa Japan; ^3^ Department of Biostatistics Yamaguchi University Graduate School of Medicine Ube Japan; ^4^ Department of Medical Oncology Osaka City General Hospital Osaka Japan; ^5^ Gastrointestinal Medical Oncology Division National Cancer Center Hospital Tokyo Japan; ^6^ Department of Medical Oncology Saku Central Hospital Advanced Care Center Saku Japan; ^7^ Department of Gastrointestinal Cancer Center Sano Hospital Kobe Japan; ^8^ Department of Internal Medicine Himeji Red Cross Hospital Himeji Japan; ^9^ Department of Medical Oncology Ichinomiyanishi Hospital Ichinomiya Japan; ^10^ Division of Gastroenterology and Hepatology, Department of Internal Medicine Jikei University School of Medicine Tokyo Japan; ^11^ Center for Clinical Research Yamaguchi University Hospital Ube Japan; ^12^ Liquid Genomics, Inc. Torrance California USA; ^13^ Department of Oncology and General Medicine Institute of Medical Science Hospital, University of Tokyo Tokyo Japan; ^14^ Department of Early Clinical Development Kyoto University Graduate School of Medicine Kyoto Japan

**Keywords:** bevacizumab, cetuximab, early tumor shrinkage, metastatic colorectal cancer

## Abstract

**Background:**

Long‐term anti‐EGFR antibody treatment increases the risk of severe dermatologic toxicities. This single‐arm, phase II trial aimed to investigate the strategy of switching from cetuximab to bevacizumab in combination with FOLFIRI based on early tumor shrinkage (ETS) in patients with *RAS* wild‐type metastatic colorectal cancer (mCRC).

**Methods:**

Radiologic assessment was performed to evaluate ETS, defined as ≥20% reduction in the sum of the largest diameters of target lesions 8 weeks after the introduction of FOLFIRI plus cetuximab. ETS‐negative patients switched to FOLFIRI plus bevacizumab, whereas ETS‐positive patients continued FOLFIRI plus cetuximab for eight more weeks, with a switch to FOLFIRI plus bevacizumab thereafter. The primary endpoint was progression‐free survival.

**Results:**

This trial was prematurely terminated due to poor accrual after a total enrollment of 30 patients. In 29 eligible patients, 7 were ETS‐negative and 22 were ETS‐positive. Two ETS‐negative patients and 17 ETS‐positive patients switched to FOLFIRI plus bevacizumab 8 weeks and 16 weeks after initial FOLFIRI plus cetuximab, respectively. Median progression‐free and overall survival durations were 13.4 and 34.7 months, respectively. Six (20%) patients experienced grade ≥3 paronychia, which improved to grade ≤2 by 18 weeks. Grade ≥3 acneiform rash, dry skin, and pruritus were not observed in any patients.

**Conclusions:**

Our novel treatment strategy delivered acceptable survival outcomes and reduced severe dermatologic toxicities.

## 1 INTRODUCTION

Optimal sequence of molecular targeted agents including antibodies against epidermal growth factor receptor (EGFR), such as cetuximab and panitumumab, and vascular endothelial growth factor, such as bevacizumab, is clinically important for the treatment of metastatic colorectal cancer (mCRC).^1^, ^2^ Anti‐EGFR antibodies in combination with doublet cytotoxic agents are recommended in first‐line chemotherapy for patients with *RAS* wild‐type and left‐sided mCRC.^3^

A remarkable benefit of adding anti‐EGFR antibody to standard first‐line chemotherapy is the ability to achieve earlier and deeper tumor response compared to that obtained with anti‐vascular endothelial growth factor antibody in patients with *RAS* wild‐type mCRC.^4^, ^5^ Early tumor shrinkage (ETS) is an on‐treatment measurement defined as a reduction in tumor size, which is the sum of the largest diameters of all target lesions, during early radiologic assessment conducted usually 6–8 weeks after treatment initiation.^6^, ^7^ ETS occurs in around 60%–70% of patients with mCRC receiving anti‐EGFR antibody‐containing first‐line chemotherapy.^7^ ETS is associated with improved progression‐free survival (PFS) and overall survival (OS) in patients with mCRC treated with first‐line chemotherapy; these associations are more pronounced in cases where cetuximab is added to chemotherapy.^7^, ^8^ Thus, ETS may be a good predictor of long‐term outcomes at individual level and may be useful in guiding on‐treatment decisions including continuation or discontinuation of cetuximab‐containing chemotherapy. Achieving deeper tumor shrinkage can increase the possibility of conversion surgery and improve post‐progression survival, leading to prolonged OS.^9^ Conversely, dermatologic toxicities such as acneiform rash, dry skin, and paronychia are common and unfavorable side effects of anti‐EGFR antibody therapy.^10^, ^11^ Cetuximab‐containing first‐line chemotherapy regimens have been reported to be more detrimental to the quality of life (QOL), in particular dermatology‐related QOL, of patients compared to bevacizumab‐containing chemotherapy regimens.^12^ Thus, considering the balance between the advantages and toxicities of anti‐EGFR antibodies is important during the care of individual patients beyond the biomarker‐stratified treatment initiation. However, current standard care allows the continuation of treatment with the same molecular targeted agent until disease progression or intolerable toxicity. Moreover, despite the evaluation of optimal maintenance treatment following anti‐EGFR antibodies plus doublet chemotherapy in recent clinical trials, preferred maintenance regimens include the continuous use of anti‐EGFR antibodies in combination with 5‐fluorouracil/leucovorin.^13^ Thus, long‐term exposure of anti‐EGFR antibodies could deteriorate QOL.

In this phase II HYBRID study, we aimed to evaluate whether switching first‐line treatment with FOLFIRI plus cetuximab to FOLFIRI plus bevacizumab based on ETS achievement would be associated with better clinical course with reduced dermatologic toxicities in patients with *RAS* wild‐type mCRC.

## 2 METHODS

### 2.1 Study design and patients

In this single‐arm, open‐label, multicenter, phase II trial, patients were recruited from 10 participating centers in Japan. The eligibility criteria were as follows: age ≥ 20 years, histologically confirmed adenocarcinoma of the colon or rectum, wild‐type *RAS*, unresectable metastatic or recurrent disease, Eastern Cooperative Oncology Group performance status score of 0–2, no previous chemotherapy for colorectal cancer excluding adjuvant therapy completed at least 6 months prior to trial enrollment, presence of target lesions according to the Response Evaluation Criteria in Solid Tumors (version 1.1), and adequate organ function. Patients with the following conditions were excluded: symptomatic brain metastasis, intestinal paralysis/obstruction, massive pleural effusion, ascites, and pericardial effusion, uncontrolled diarrhea, grade ≥2 dermatologic toxicity, interstitial pneumonia, or pulmonary fibrosis, history of thromboembolism, and unhealed wounds. The trial was conducted in accordance with the protocol and in compliance with the Declaration of Helsinki. The protocol was approved by the ethics committees of St. Marianna University School of Medicine, Osaka City General Hospital, National Cancer Center Hospital, Saku Central Hospital Advanced Care Center, Sano Hospital, Himeji Red Cross Hospital, Shikoku Cancer Center, and Jikei University School of Medicine. All patients provided written informed consent before trial entry. This trial was registered in the University Hospital Medical Information Network Clinical Trials Registry (UMIN000023026).

### 2.2 Procedures

**FIGURE 1:**
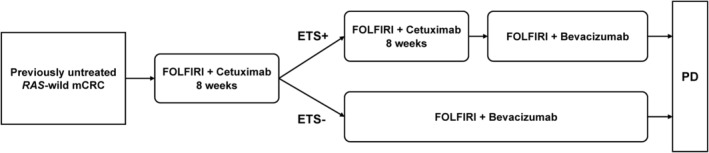
Treatment schema of the HYBRID trial. Enrolled patients received 8‐week induction chemotherapy with FOLFIRI plus cetuximab. ETS was assessed at 8 weeks. ETS‐negative patients immediately switched to FOLFIRI plus bevacizumab. ETS‐positive patients further received 8‐week chemotherapy with FOLFIRI plus cetuximab and thereafter switched to FOLFIRI plus bevacizumab at 16 weeks. ETS, early tumor shrinkage; mCRC, metastatic colorectal cancer; PD, progressive disease.

Enrolled patients were initially treated with FOLFIRI plus cetuximab. Radiologic assessment was performed 8 weeks after treatment initiation to evaluate ETS, defined as ≥20% reduction in the sum of the largest diameters of all target lesions. Patients who did not achieve ETS were immediately switched to FOLFIRI plus bevacizumab. Patients who achieved ETS continued FOLFIRI plus cetuximab for a total of 16 weeks after treatment initiation, followed by switch to FOLFIRI plus bevacizumab. Figure 1 shows the study schema.

Treatment schedule and dosages were as follows. Cetuximab was administered weekly at an initial dose of 400 mg/m^2^ thereafter 250 mg/m^2^; bevacizumab was administered biweekly at a dose of 5 mg/kg; FOLFIRI comprised 180 mg/m^2^ irinotecan and 5‐fluorouracil with 400 mg/m^2^ bolus followed by 2400 mg/m^2^ continuous infusion over 46 h, administered every 2 weeks. The dose of irinotecan was reduced to 150 mg/m^2^ in patients harboring *UGT1A1* *28/*28, *6/*6, or *28/*6 polymorphisms. Dermatologic toxicity management was performed by preemptive administration of skin moisturizer and minocycline and the reactive dosing of topical steroid. The protocol treatment was continued until disease progression, emergence of unacceptable toxicities, conversion surgery, patient refusal of treatment, or physician's decision to discontinue treatment.

Tumor response was assessed by computed tomography or magnetic resonance imaging studies conducted every two months according to the Response Evaluation Criteria in Solid Tumors (version 1.1). Blinded independent central review was not conducted. Adverse events were continuously monitored during the protocol treatment according to the National Cancer Institute Common Terminology Criteria for Adverse Events (version 4.0).

### 2.3 Biomarker measurement

The correlation of ETS with mutations in circulating tumor DNA (ctDNA) was examined. To this end, plasma samples were collected immediately before the initiation of protocol treatment. Free‐circulating DNA was isolated using the QIAamp® Circulating Nucleic Acid kit (Qiagen) according to the manufacturer's instructions. Target mutations in *KRAS*, *NRAS*, *PIK3CA*, and *BRAF* were evaluated using competitive allele‐specific TaqMan® polymerase chain reaction assays (Life Technologies). Mutant allele fractions were calculated using standard curves generated from dilutions of positive controls with known quantities of mutant over wild‐type DNA with polymerase chain reaction cycle thresholds.

### 2.4 Statistical analysis

The primary endpoint was PFS, defined as the time from the date of enrollment to the first documentation of disease progression or death from any cause. Secondary endpoints were objective response rate, OS, depth of response (DpR, defined as maximum percent change in tumor size compared with baseline), and safety. We hypothesized that the treatment strategy of switching from cetuximab to bevacizumab would prolong PFS. Based on previous studies on standard first‐line chemotherapeutic regimens for mCRC, we determined 10 months as the threshold median PFS. We assumed a 5‐month improvement of median PFS with the switch from cetuximab to bevacizumab and therefore set 15 months as the expected value. With a one‐sided α of 10% and power of 70%, 51 patients were required. Considering an approximate dropout rate of 5%, 54 patients were planned for enrollment. The Kaplan–Meier method with the log‐rank test was used to estimate and compare survival outcomes between patients with and without ETS. Analyses were two‐sided at a significance level of 0.05 and were performed using SAS 9.4 (SAS Institute, Cary, NC, USA).

## 3 RESULTS

### 3.1 Patients

A total of 30 patients were enrolled between October 2016 and June 2018, and the trial was prematurely terminated due to poor accrual. One patient with primary tumor in the appendix was ineligible. Therefore, treatment efficacy was evaluated in 29 eligible patients (full analysis set) with a median follow‐up of 25.4 months. Safety was evaluated for all 30 patients (safety analysis set) (Figure S1).

Table 1 summarizes the baseline characteristics of 29 eligible patients. Median age was 66 (range, 53–78) years, and 21 (72%) patients were male. Majority of the patients had left‐sided primary tumors (72%) and liver metastases (86%). No patients had received adjuvant chemotherapy.

**TABLE 1:** Patient characteristics.

Characteristics	*N* = 29
Age	
Median (range)	66 (53–78)
<65	11 (38%)
≥65	18 (62%)
Sex	
Male	21 (72%)
Female	8 (28%)
ECOG performance status	
0	21 (72%)
1	7 (24%)
2	1 (3%)
Primary tumor location	
Right‐sided	8 (28%)
Lest‐sided	21 (72%)
Number of metastatic sites	
1	17 (59%)
≥ 2	12 (41%)
Liver metastases	
Present	25 (86%)
Absent	4 (14%)
Adjuvant chemotherapy	
No	29 (100%)
Yes	0 (0%)
*UGT1A1* genotype	
*1/*1	15 (52%)
*1/*28 or *1/*6	12 (41%)
*28/*28, *6/*6, or *28/*6	2 (7%)

Abbreviation: ECOG, Eastern Cooperative Oncology Group.

### 3.2 Efficacy

Among the 29 eligible patients, objective response, and disease control rates were 72.4% and 89.7%, respectively. Median DpR was 47.7%, and median time to DpR was 3.9 months. Of these 29 patients, 22 (75.9%) achieved ETS (Table 2, Figure S1). Median time to DpR was 5.4 months in patients with ETS and 1.9 months in those without ETS (Table 2).

**TABLE 2:** Summary of efficacy.

Outcome	Results (*N* = 29)
Median PFS	13.4 months
80% CI	10.3–14.5
95% CI	9.0–15.8
Median OS	34.7 months
95% CI	24.2‐NR
Tumor response	
CR	1 (3.4%)
PR	20 (69.0%)
SD	5 (17.2%)
PD	3 (10.3%)
Overall response rate	72.4%
Disease control rate	89.7%
ETS	
Positive	22 (75.9%)
Negative	7 (24.1%)
Median DpR	
All patients	47.7%
ETS‐positive patients (*N* = 22)	60.7%
ETS‐negative patients (*N* = 7)	9.0%
Median time to DpR	
All patients	3.9 months
ETS‐positive patients (*N* = 22)	5.4 months
ETS‐negative patients (*N* = 7)	1.9 months

Abbreviations: CI, confidence interval; CR, complete response; DpR, depth of response; ETS, early tumor shrinkage; OS, overall survival; PD, progressive disease; PFS, progression‐free survival; PR, partial response; SD, stable disease.

Among the 22 patients who achieved ETS, 17 patients switched to FOLFIRI plus bevacizumab at 16 weeks. The remaining five patients with ETS did not switch to FOLFIRI plus bevacizumab because of conversion surgery (*n* = 4) or disease progression (*n* = 1) which occurred during the first 8 weeks following induction treatment. Only two of the seven patients without ETS switched to FOLFIRI plus bevacizumab at 8 weeks, whereas the remaining five patients without ETS could not switch to FOLFIRI plus bevacizumab because of toxicities (*n* = 3, including infusion reaction by cetuximab, infection of implantable central venous access port, and aspiration pneumonia in one patient each), early disease progression (*n* = 1), and patient request (*n* = 1) (Figure S1). These five patients subsequently received FOLFOX (*n* = 2), FOLFOX plus bevacizumab (*n* = 1), FOLFOX plus panitumumab (*n* = 1), and best supportive care (*n* = 1); the last patient developed aspiration pneumonia while receiving FOLFIRI plus cetuximab.

**FIGURE 2:**
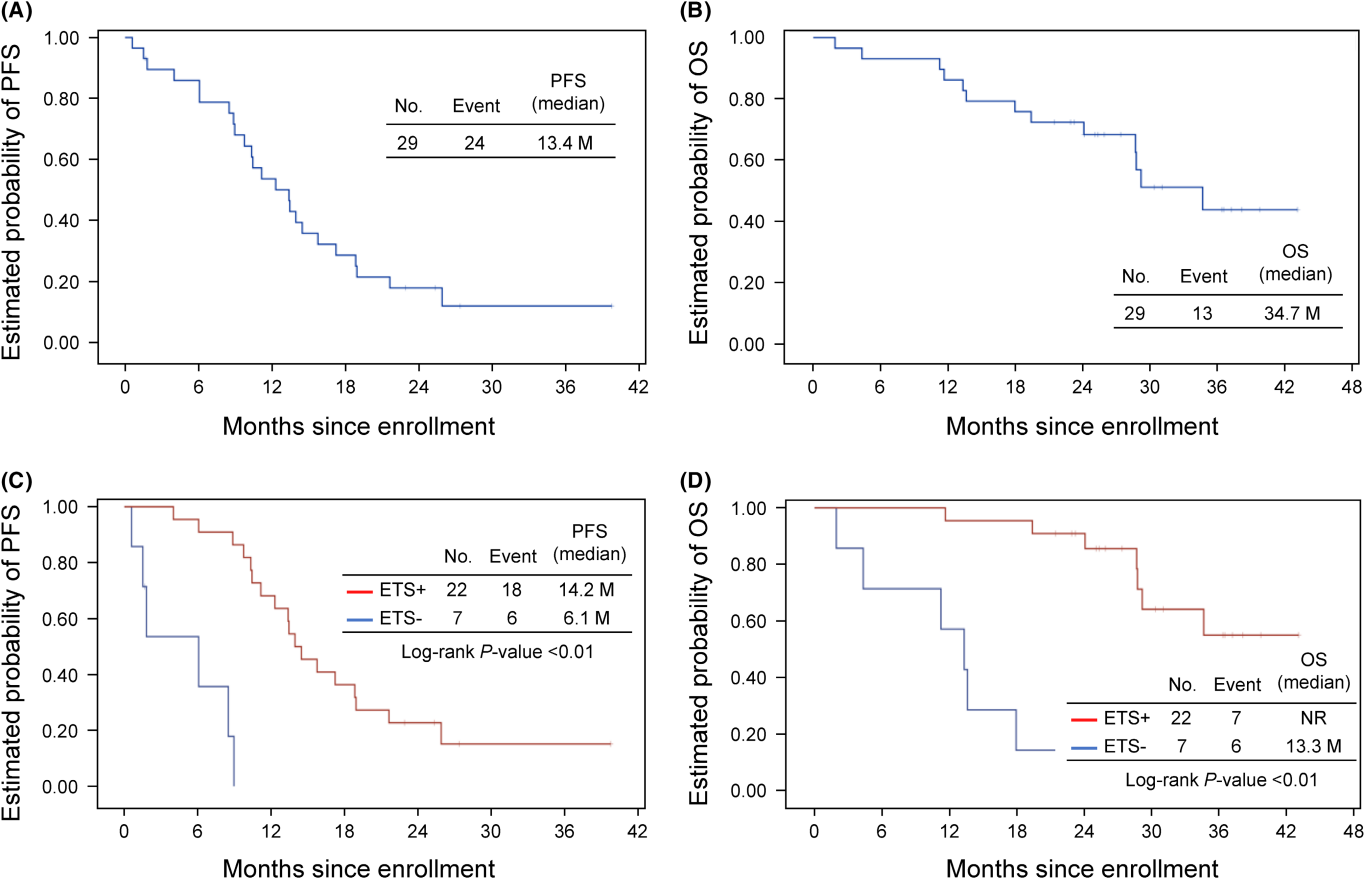
Kaplan–Meier curves show (A) PFS in eligible patients, (B) OS in eligible patients, (C) PFS in ETS‐positive and ETS‐negative patients, and (D) OS in ETS‐positive and ETS‐negative patients. ETS, early tumor shrinkage; NR, not reached; OS, overall survival; PFS, progression‐free survival.

Median PFS and OS were 13.4 months (80% confidence interval [CI] 10.3–14.5 months) and 34.7 months (95% CI 24.2 months–not reached), respectively. Patients with ETS had significantly longer PFS (median, 14.2 vs. 6.1 months, *p* < 0.01) and OS (median, not reached vs. 13.3 months, *p* < 0.01) than those without ETS (Figure 2; Table 2). In two patients without ETS who switched to FOLFIRI plus bevacizumab at 8 weeks, PFS and OS were 9.1 and 18.2 months, respectively, in one patient, and 8.6 and 13.5 months, respectively, in the other patient.

### 3.3 Safety

**FIGURE 3:**
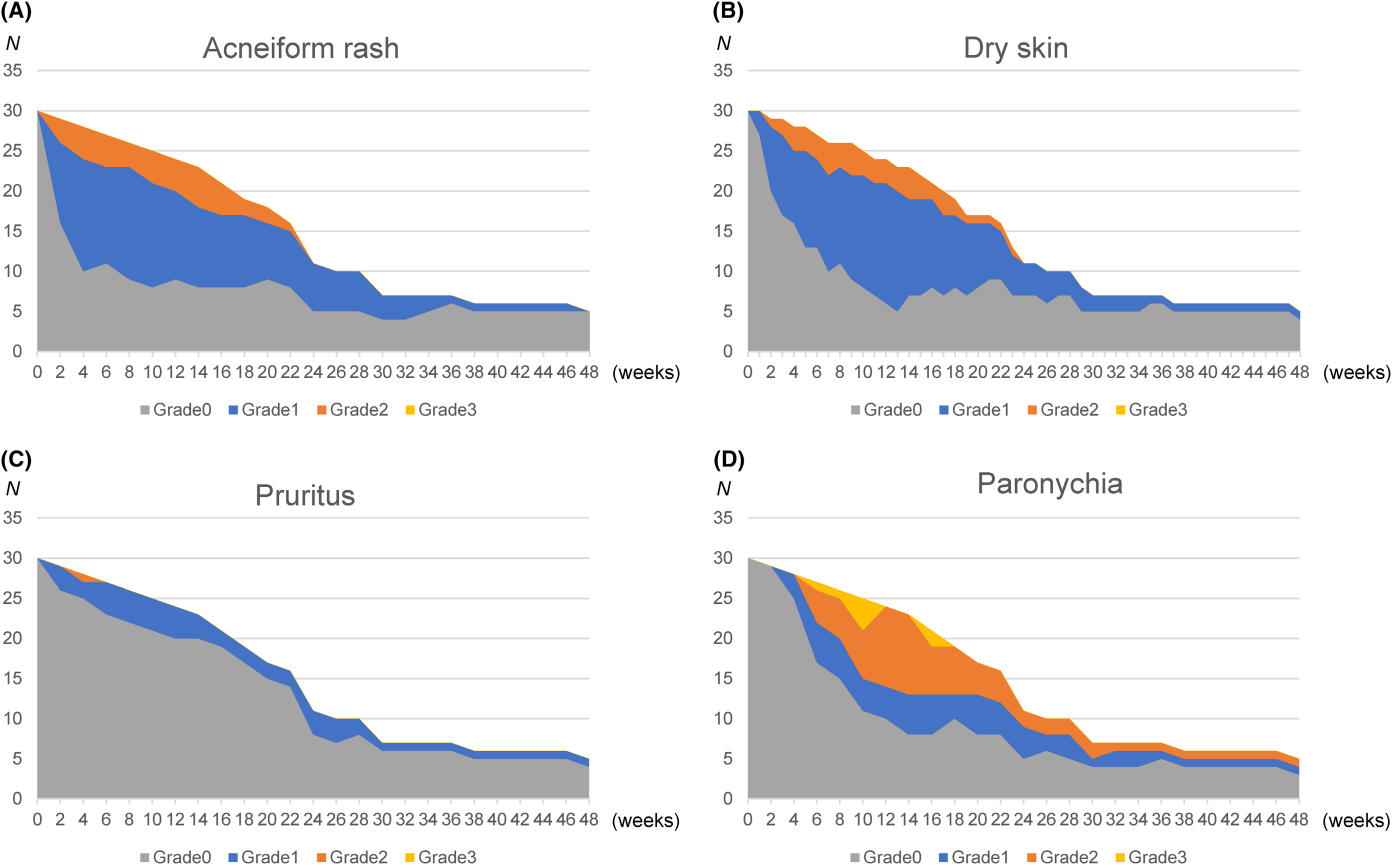
Temporal changes in the distribution of number of patients with specific grades of cetuximab‐related dermatologic toxicities. (A) Acneiform rash, (B) dry skin, (C) pruritus, and (D) paronychia. Vertical axes show patient number, and horizontal axes show time from initiation of protocol treatment.

The most frequent grade ≥3 adverse event was neutropenia (60%). Six (20%) patients developed grade ≥3 paronychia, whereas other grade ≥3 dermatologic toxicities (acneiform rash, dry skin, or pruritus) were not noted (Table 3). Five patients with ETS developed grade ≥3 paronychia at a median of 11 weeks (range, 5–16 weeks), whereas one patient without ETS developed grade ≥3 paronychia at 7 weeks. All grade ≥3 paronychia improved to grade ≤2 by 18 weeks after treatment initiation (Figure 3A). The frequencies of grade 2 acneiform rash, dry skin, and pruritus were 20%, 20%, and 7%, respectively (Table 3). Grade 2 acneiform rash and dry skin improved to grade 1 or 0 by 24 weeks after treatment initiation, and grade 2 pruritus improved to grade 1 or 0 by 6 weeks after treatment initiation (Figure 3B–D). The rate of major adverse events did not increase after the switch, except for grade ≥3 hypertension (Table S1).

**TABLE 3:** Adverse events.

Adverse event	*N* = 30					
	Grade 1 *N* (%)	Grade 2 *N* (%)	Grade 3 *N* (%)	Grade 4 *N* (%)	All grade *N* (%)	Grade ≥3 *N* (%)
Leucopenia	3 (10)	14 (47)	5 (17)	1 (3)	23 (77)	6 (20)
Neutropenia	0 (0)	6 (20)	14 (47)	4 (13)	24 (80)	18 (60)
Anemia	6 (20)	7 (23)	0 (0)	0 (0)	13 (43)	0 (0)
Thrombocytopenia	14 (47)	0 (0)	0 (0)	0 (0)	14 (47)	0 (0)
Hypomagnesemia	15 (50)	1 (3)	1 (3)	0 (0)	17 (57)	1 (3)
Anorexia	9 (30)	5 (17)	2 (7)	0 (0)	16 (53)	2 (7)
Nausea	12 (40)	2 (7)	1 (3)	0 (0)	15 (50)	1 (3)
Diarrhea	8 (27)	7 (23)	2(7)	0 (0)	17 (57)	2 (7)
Paronychia	5 (17)	7 (23)	6 (20)	0 (0)	18 (60)	6 (20)
Acneiform rash	20 (67)	6 (20)	0 (0)	0 (0)	26 (87)	0 (0)
Dry skin	18 (60)	6 (20)	0 (0)	0 (0)	24 (80)	0 (0)
Pruritus	6 (20)	2 (7)	0 (0)	0 (0)	8 (27)	0 (0)
Hypertension	4 (13)	9 (30)	2 (7)	0 (0)	15 (50)	2 (7)
Proteinuria	11 (37)	4 (13)	0 (0)	0 (0)	15 (50)	0 (0)

### 3.4 Biomarkers

Plasma samples at the time of pretreatment were available for biomarker analysis in 28 patients. Target sequencing using ctDNA revealed that 4 (14.3%) patients harbored *RAS* or *BRAF* mutations, including 1 (3.6%) patient with the *KRAS* G13D mutation and 3 (10.7%) patients with the *BRAF* V600E mutation (Table S2). None of these four patients achieved ETS. Conversely, the *PIK3CA* E545K mutation was detected in 2 (7.1%) patients, both of whom achieved ETS (Table S3). After excluding the 4 patients with *RAS* or *BRAF* mutations detected in ctDNA, ETS was achieved in 22 of the 25 patients (88%). In these 25 patients without *RAS* or *BRAF* mutations, median OS was not reached and median PFS was 13.7 months.

## 4 DISCUSSION

This was the first study to prospectively investigate the treatment strategy of switching from FOLFIRI plus cetuximab to FOLFIRI plus bevacizumab based on ETS during first‐line treatment in patients with *RAS* wild‐type mCRC. This novel strategy led to acceptable survival outcomes and reduced rates of severe dermatologic toxicities. Despite the early termination of trial enrollment, a sufficient observation period was ensured to evaluate the treatment outcomes and occurrence of dermatologic toxicities in enrolled patients. The results support the clinical application of this treatment strategy pending further validation studies.

In the present trial, the treatment strategy was based on two expectations. First, we expected that survival would improve with an early switch to FOLFIRI plus bevacizumab in patients failing ETS during initial treatment with FOLFIRI plus cetuximab, as these patients would not be likely to benefit from the continuation of cetuximab‐based chemotherapy.^6^ Second, we expected this approach would prevent severe dermatologic toxicities caused by long‐term cetuximab exposure of more than 16 weeks in patients achieving ETS. In the FIRE‐3 trial, the median time to response was approximately 5 months in patients treated with FOLFIRI plus cetuximab.^9^ In the present study, the median time to DpR, which was 3.9 months in all patients, was better in those who achieved ETS than in those who failed to achieve ETS (5.4 vs. 1.9 months). This result suggested that switching from cetuximab to bevacizumab at 16 weeks in patients with ETS and at 8 weeks in those without ETS ensured maximum tumor shrinkage effect of initial FLOFIRI plus cetuximab regardless of the ETS in most patients. On the other hand, in the J‐STEPP trial investigating the management of dermatologic toxicities of panitumumab in Japanese patients with mCRC, the cumulative incidence of grade ≥2 dermatologic toxicities during the first 12 weeks of treatment was 42.6% in patients receiving preemptive skin treatment.^14^ In the present study, grade 3 paronychia emerged at a median of 11 weeks (range, 5–16 weeks) in patients with ETS, whereas at 7 weeks in a patient without ETS. Thus, the timing of the switch from cetuximab to bevacizumab in this study might be considered appropriate for preventing severe dermatologic toxicities especially in patients without ETS.

Our novel treatment strategy exhibited acceptable efficacy in terms of both PFS (median, 13.4 months) and OS (median, 34.7 months). Unexpectedly, only two patients without ETS could switch to FOLFIRI plus bevacizumab at 8 weeks. Thus, it was not possible to determine if our treatment strategy was beneficial for patients who did not achieve ETS. Conversely, 17 of the 22 patients with ETS (77%) switched to FOLFIRI plus bevacizumab at 16 weeks. The median PFS of 14.2 months in these patients with ETS was relatively longer than that of the patients achieving ETS after FOLFIRI plus cetuximab in the FIRE‐3 trial (median, 9.7 months).^9^ Thus, it is conceivable that the switch to FOLFIRI plus bevacizumab at 16 weeks might not compromise the long‐term efficacy of chemotherapy, at least in patients with ETS.

As expected, our treatment strategy reduced the severity of cetuximab‐induced dermatologic toxicities. The protocol of the current trial prespecified modified preemptive skin management, including the preemptive administration of skin moisturizer and minocycline and the reactive dosing of topical steroids, which were less proactive than the preemptive management utilized in the STEPP and J‐STEPP trials.^14^, ^15^ However, the early switch from cetuximab to bevacizumab completely prevented grade ≥3 acneiform rash, dry skin, and pruritus. Even though grade ≥3 paronychia emerged in 20% of patients, it improved to grade ≤2 by 18 weeks. Moreover, our strategy successfully prevented all grade ≥2 dermatologic toxicities 24 weeks after treatment initiation. These results indicate that strategically avoiding long‐term cetuximab exposure can mitigate the severity and duration of dermatologic toxicities and preserve the QOL of patients.

A recent phase III trial (FIRE‐4) reported that an early switch after 8–12 cycles from FOLFIRI plus cetuximab to maintenance therapy with 5‐fluorouracil/capecitabine plus bevacizumab was not associated with superior efficacy compared to the continued cetuximab administration.^16^ However, no detrimental effects were reported in patients who switched to bevacizumab. The treatment approach evaluated in FIRE‐4 was different from that in the present trial in several aspects, including the timing of the switch and the strength of backbone chemotherapy after the switch to bevacizumab. Upon detailed information of safety profiles in the FIRE‐4, especially those related to dermatologic toxicities, the clinical implication of early cetuximab‐bevacizumab switch could be more discussed.

The exploratory analysis using ctDNA revealed that four (14.3%) patients harbored *RAS* or *BRAF* mutations, including one patient with *KRAS* mutation that was not detected during the assessment of the tumor tissue. None of these four patients achieved ETS, whereas most of the other patients who did not have either *RAS* or *BRAF* mutations by ctDNA assessment achieved ETS and experienced favorable OS and PFS. These results highlight the utility of liquid biopsy as a complementary approach for tissue‐based analysis and suggest it could enrich ETS‐positive patients in treatment using anti‐EGFR antibody.

The present study has two notable limitations. First, the planned patient accrual could not be completed. Thus, the findings remain exploratory due to the insufficient statistical power and warrant further validation studies. To implement our treatment options in clinical practice, phase III trials are required. We believe that this treatment approach deserves additional validation through such confirmatory trials in the future. Second, the present trial allowed the enrollment of patients with left‐sided and right‐sided primary tumors. Furthermore, in this trial, confirmation of the *BRAF* status was not a mandatory requirement before enrollment. These conditions differ from the current treatment approaches where the first‐line use of anti‐EGFR antibodies is only recommended in patients with *RAS/BRAF* wild‐type and left‐sided tumors.^17^

In summary, the potential benefits of the novel first‐line treatment strategy, involving an early switch from FOLFIRI plus cetuximab to FOLFIRI plus bevacizumab based on ETS, may offer acceptable efficacy and potentially reduce cetuximab‐induced dermatologic toxicities in patients with *RAS* wild‐type mCRC. However, it is important to note that the clinical applicability of this strategy necessitates further validation studies.

## AUTHOR CONTRIBUTIONS

**Hiroyuki Arai:** Visualization (lead); writing – original draft (lead). **Takashi Tsuda:** Conceptualization (lead); methodology (lead). **Yu Sunakawa:** Project administration (supporting); supervision (supporting); writing – review and editing (equal). **Mototsugu Shimokawa:** Data curation (lead); formal analysis (lead); software (lead); visualization (supporting); writing – review and editing (supporting). **Kohei Akiyoshi:** Investigation (equal); resources (equal); writing – review and editing (equal). **Shinya Tokunaga:** Investigation (equal); resources (equal); writing – review and editing (equal). **Hirokazu Shoji:** Investigation (equal); resources (equal); writing – review and editing (equal). **Kenji Kunieda:** Investigation (equal); resources (equal); writing – review and editing (equal). **Masahito Kotaka:** Investigation (equal); resources (equal); writing – review and editing (equal). **Toshihiko Matsumoto:** Investigation (equal); resources (equal); writing – review and editing (equal). **Yusuke Nagata:** Investigation (equal); resources (equal); writing – review and editing (equal). **Takuro Mizukami:** Investigation (equal); resources (equal); writing – review and editing (equal). **Fumitaka Mizuki:** Investigation (equal); resources (equal); writing – review and editing (equal). **Kathleen D. Danenberg:** Resources (equal); writing – review and editing (equal). **Narikazu Boku:** Investigation (equal); resources (equal); writing – review and editing (equal). **Takako Eguchi Nakajima:** Project administration (lead); supervision (lead); writing – review and editing (lead).

## FUNDING INFORMATION

We had no financial supports for this study.

## CONFLICT OF INTEREST STATEMENT

YS reports grants or contracts from Chugai Pharmaceutical, Taiho Pharmaceutical, Takeda, Sanofi, Ohtsuka Pharmaceutical, and Eli Lilly Japan, honoraria from Eli Lilly Japan, Bristol‐Byers Squibb, Chugai Pharmaceutical, Takeda, Ono Pharmaceutical, Merck Biopharma, Taiho Pharmaceutical, Bayer, Daiichi‐Sankyo, MSD, Sysmex, and Guardant Health, and advisory sole for Merck Biopharma, Ono Pharmaceutical, and Guardant Health; TM1 reports research funding from Ono Pharmaceutical Co, Ltd and Sanofi Co, Ltd, honoraria from Bayer Co, Ltd, Bristol‐Myers Squibb Co, Ltd, Chugai Pharmaceutical Co, Ltd, Daiichi Sankyo Co, Ltd, Eli Lilly Japan Co, Ltd, Merck Bio Pharma Co, Ltd, MSD Co, Ltd, Ono Pharmaceutical Co, Ltd, Sanofi Co, Ltd, Taiho Pharmaceutical Co, Ltd, Takeda Co, Ltd, Teijin Pharmaceutical Co, Ltd, and Yakult Honsha Co, Ltd; NB reports honoraria from Ono, Taiho, Daiichi‐Sankyo, Eli‐Lilly, and Bristol, and research fund from Ono and Takeda; TEN reports grants or contracts from Guardant Health, Taiho Pharmaceutical Co, Takeda Pharmaceutical Co, Chugai Pharmaceutical Co, Nippon Kayaku Co, AbbVie, Eli Lilly Japan K.K., Shionogi Pharmaceutical Co, Otsuka Pharmaceutical Co, and Taisho Pharmaceutical Co, and honoraria from Sumitomo Dainippon Pharma Co, Boehringer Ingelheim, Bristol‐Myers S1uibb, Ono Pharmaceutical Co, Taiho Pharmaceutical Co, Amgen, Takeda Pharmaceutical Co, Chugai Pharmaceutical Co, Sanofi K.K., Novartis Japan, Daiichi Sankyo Co, AstraZeneca, IQVIA, GlaxoSmithKline, NOBEL Pharma, and Parexel. All remaining authors have declared no conflicts of interest.

## ETHICS STATEMENT

The trial was conducted in accordance with the protocol and in compliance with the Declaration of Helsinki. The protocol was approved by the ethics committees of all participating centers. All patients provided written informed consent before trial entry.

## CLINICAL TRIAL REGISTRATION NUMBER

This trial was registered in the University Hospital Medical Information Network Clinical Trials Registry (UMIN000023026).

## Supporting information


Figure S1.

Table S1.

Table S2.

Table S3.

## Data Availability

The data sets used and analyzed during this study are available from the corresponding author on reasonable request.
