# Supplementary material for: Switching from FOLFIRI plus cetuximab to FOLFIRI plus bevacizumab based on early tumor shrinkage in RAS wild‐type metastatic colorectal cancer: A phase II trial (HYBRID)
Source: Cancer Med. 2024 Apr 9;13(7):e7107. doi: 10.1002/cam4.7107 (PMC11002633; doi:10.1002/cam4.7107)
Supplement: Supplementary file 1 — Figure S1. [file CAM4-13-e7107-s003.pdf]

Figure S1

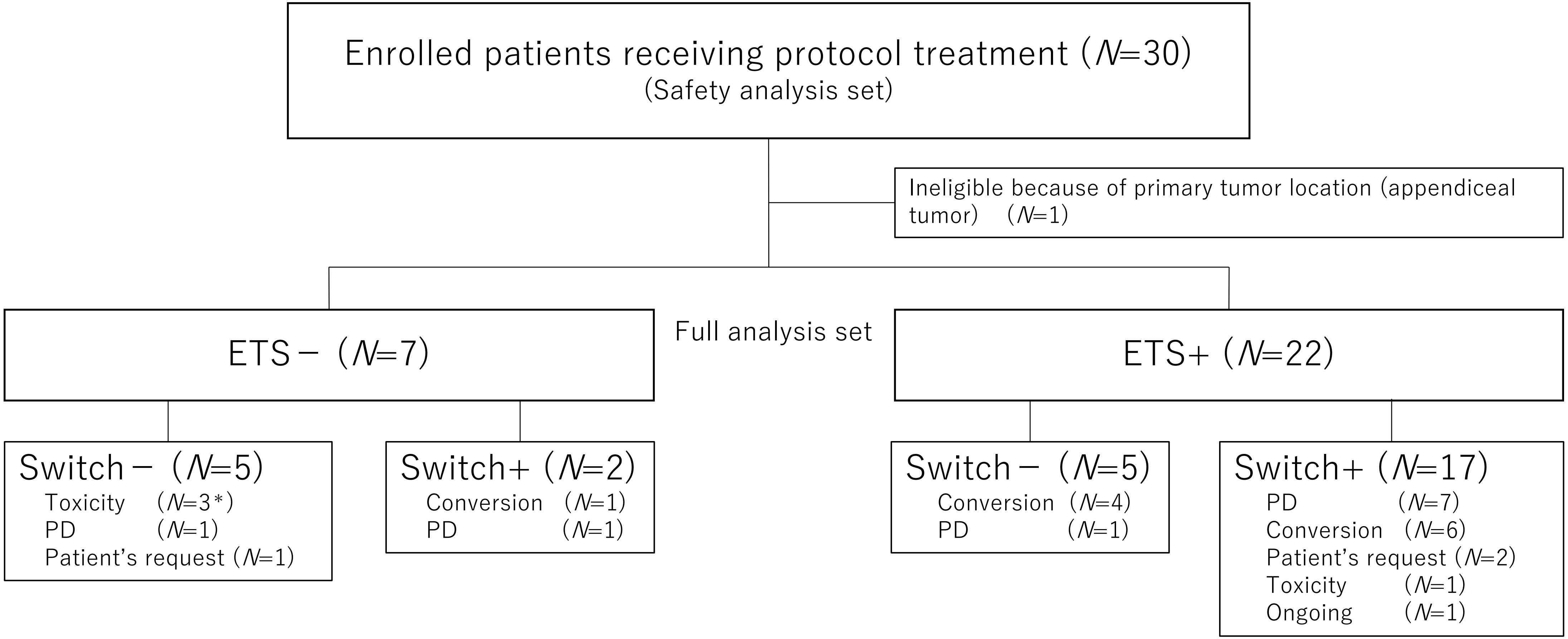

CONSORT diagram.

* Toxicities included grade 3 of infusion reaction by cetuximab, grade 3 of infection of implantable central venous access port, and grade 3 of aspiration pneumonia

Abbreviations: ETS, early tumor shrinkage; PD, progressive disease.
