# Supplementary material for: Switching from FOLFIRI plus cetuximab to FOLFIRI plus bevacizumab based on early tumor shrinkage in RAS wild‐type metastatic colorectal cancer: A phase II trial (HYBRID)
Source: Cancer Med. 2024 Apr 9;13(7):e7107. doi: 10.1002/cam4.7107 (PMC11002633; doi:10.1002/cam4.7107)
Supplement: Supplementary file 2 — Table S1. [file CAM4-13-e7107-s001.docx]

**Table S1. Adverse events before and after switching from FOLFIRI plus cetuximab to FOLFIRI plus bevacizumab**

| Adverse event | |  | | | | | | | | | | | |
| --- | --- | --- | --- | --- | --- | --- | --- | --- | --- | --- | --- | --- | --- |
|  |  | Grade 1  *N* (%) | | Grade 2  *N* (%) | | Grade 3  *N* (%) | | Grade 4  *N* (%) | | All grade  *N* (%) | | Grade > 3  *N* (%) | |
| Leucopenia | Cetuximab (n=29) | 2 | (7) | 14 | (48) | 4 | (14) | 1 | (3) | 21 | (72) | 5 | (17) |
|  | Bevacizumab (n=19) | 1 | (5) | 5 | (26) | 1 | (5) | 0 | (0) | 7 | (37) | 1 | (5) |
| Neutropenia | Cetuximab (n=29) | 0 | (0) | 7 | (24) | 12 | (41) | 4 | (14) | 23 | (79) | 16 | (55) |
|  | Bevacizumab (n=19) | 1 | (5) | 6 | (32) | 4 | (21) | 0 | (0) | 11 | (58) | 4 | (21) |
| Anemia | Cetuximab (n=29) | 5 | (17) | 6 | (21) | 0 | (0) | 0 | (0) | 11 | (38) | 0 | (0) |
|  | Bevacizumab (n=19) | 3 | (16) | 4 | (21) | 0 | (0) | 0 | (0) | 7 | (37) | 0 | (0) |
| Thrombocytopenia | Cetuximab (n=29) | 13 | (45) | 0 | (0) | 0 | (0) | 0 | (0) | 13 | (45) | 0 | (0) |
|  | Bevacizumab (n=19) | 5 | (26) | 0 | (0) | 0 | (0) | 0 | (0) | 5 | (26) | 0 | (0) |
| Hypomagnesemia | Cetuximab (n=29) | 12 | (41) | 1 | (3) | 1 | (3) | 0 | (0) | 14 | (48) | 1 | (3) |
|  | Bevacizumab (n=19) | 8 | (42) | 1 | (5) | 1 | (5) | 0 | (0) | 10 | (53) | 1 | (5) |
| Anorexia | Cetuximab (n=29) | 8 | (28) | 5 | (17) | 2 | (7) | 0 | (0) | 15 | (52) | 2 | (7) |
|  | Bevacizumab (n=19) | 3 | (16) | 0 | (0) | 1 | (5) | 0 | (0) | 4 | (21) | 1 | (5) |
| Nausea | Cetuximab (n=29) | 11 | (38) | 2 | (7) | 1 | (3) | 0 | (0) | 14 | (48) | 1 | (3) |
|  | Bevacizumab (n=19) | 2 | (11) | 0 | (0) | 0 | (0) | 0 | (0) | 2 | (11) | 0 | (0) |
| Diarrhea | Cetuximab (n=29) | 6 | (21) | 6 | (21) | 2 | (7) | 0 | (0) | 14 | (48) | 2 | (7) |
|  | Bevacizumab (n=19) | 7 | (37) | 0 | (0) | 0 | (0) | 0 | (0) | 7 | (37) | 0 | (0) |
| Paronychia | Cetuximab (n=29) | 5 | (17) | 7 | (24) | 5 | (17) | 0 | (0) | 17 | (59) | 5 | (17) |
|  | Bevacizumab (n=19) | 3 | (16) | 4 | (21) | 1 | (5) | 0 | (0) | 8 | (42) | 1 | (5) |
| Acneiform rash | Cetuximab (n=29) | 19 | (66) | 6 | (21) | 0 | (0) | 0 | (0) | 25 | (86) | 0 | (0) |
|  | Bevacizumab (n=19) | 10 | (53) | 1 | (5) | 0 | (0) | 0 | (0) | 11 | (58) | 0 | (0) |
| Dry skin | Cetuximab (n=29) | 18 | (62) | 6 | (21) | 0 | (0) | 0 | (0) | 24 | (83) | 0 | (0) |
|  | Bevacizumab (n=19) | 11 | (58) | 2 | (11) | 0 | (0) | 0 | (0) | 13 | (68) | 0 | (0) |
| Pruritus | Cetuximab (n=29) | 5 | (17) | 2 | (7) | 0 | (0) | 0 | (0) | 7 | (24) | 0 | (0) |
|  | Bevacizumab (n=19) | 3 | (16) | 0 | (0) | 0 | (0) | 0 | (0) | 3 | (16) | 0 | (0) |
| Hypertension | Cetuximab (n=29) | 7 | (24) | 8 | (28) | 1 | (3) | 0 | (0) | 16 | (66) | 1 | (3) |
|  | Bevacizumab (n=19) | 4 | (21) | 6 | (32) | 3 | (16) | 0 | (0) | 13 | (68) | 3 | (16) |
| Proteinuria | Cetuximab (n=29) | 11 | (38) | 3 | (10) | 0 | (0) | 0 | (0) | 14 | (48) | 0 | (0) |
|  | Bevacizumab (n=19) | 6 | (32) | 2 | (11) | 0 | (0) | 0 | (0) | 8 | (42) | 0 | (0) |
