# Supplementary material for: Switching from FOLFIRI plus cetuximab to FOLFIRI plus bevacizumab based on early tumor shrinkage in RAS wild‐type metastatic colorectal cancer: A phase II trial (HYBRID)
Source: Cancer Med. 2024 Apr 9;13(7):e7107. doi: 10.1002/cam4.7107 (PMC11002633; doi:10.1002/cam4.7107)
Supplement: Supplementary file 3 — Table S2. [file CAM4-13-e7107-s002.docx]

**Table S2. Mutations detected by pre-treatment ctDNA-based target sequence**

| Mutation | *N* = 28 | |
| --- | --- | --- |
| *KRAS* G13D | 1 | (3.6%) |
| *BRAF* V600E | 3 | (10.7%) |
| *PIK3CA* E545K | 2 | (7.1%) |

No duplicates.

Abbreviations: ctDNA, circulating tumor DNA.
