# Supplementary material for: Switching from FOLFIRI plus cetuximab to FOLFIRI plus bevacizumab based on early tumor shrinkage in RAS wild‐type metastatic colorectal cancer: A phase II trial (HYBRID)
Source: Cancer Med. 2024 Apr 9;13(7):e7107. doi: 10.1002/cam4.7107 (PMC11002633; doi:10.1002/cam4.7107)
Supplement: Supplementary file 4 — Table S3. [file CAM4-13-e7107-s004.docx]

**Table S3. Correlation between ctDNA-detected mutations and ETS**

| Mutation | *N* | ETS-positive | ETS-negative | ETS-positive rate |
| --- | --- | --- | --- | --- |
| *KRAS* G13D | 1 | 0 | 1 | 0% |
| *BRAF* V600E | 3 | 0 | 3 | 0% |
| *PIK3CA* E545K | 2 | 2 | 0 | 100% |

Abbreviations: ctDNA, circulating tumor DNA; ETS, early tumor shrinkage.
